# Risk factors and nomogram prediction model for checkpoint inhibitor-related pneumonitis in patients with advanced non-small cell lung cancer

**DOI:** 10.3389/fmed.2026.1742594

**Published:** 2026-03-25

**Authors:** Ya-Fang Chen, Jiao Tian, Xiao-Mei Liu, Ying Hu, Yi He

**Affiliations:** Department of Oncology Three Wards, First Affiliated Hospital of Military Medical University, Chongqing, China

**Keywords:** advanced non-small cell lung cancer, checkpoint inhibitor-related pneumonitis, immune checkpoint inhibitors, nomogram, risk factors

## Abstract

**Background:**

Immune checkpoint inhibitors have improved outcomes in advanced non-small cell lung cancer (NSCLC) but are associated with immune-related adverse events such as pneumonitis. This study aimed to identify risk factors for checkpoint inhibitor-related pneumonitis (CIP) and to develop a predictive nomogram for individualized risk assessment in advanced NSCLC patients.

**Methods:**

This retrospective study included consecutive patients with advanced NSCLC treated with ICIs between January 2021 and December 2024. All patients who developed CIP were included as cases (*n* = 96), and non-CIP controls were selected from the source population using propensity score matching (*n* = 191). CIP was diagnosed using a standardized adjudication process with systematic exclusion of infectious, malignant, radiation-related, and cardiogenic etiologies. Multivariate logistic regression was performed to identify independent predictors, and a nomogram was constructed. Model performance was evaluated using receiver operating characteristic analysis, calibration curves, bootstrap internal validation, and decision curve analysis.

**Results:**

Disease duration (OR 1.66, 95% CI 1.20–2.31), smoking history (OR 3.32, 95% CI 1.34–8.26), prior chest radiotherapy (OR 2.75, 95% CI 1.09–6.92), and baseline Hamilton Anxiety Rating Scale score (OR 1.12 per point, 95% CI 1.04–1.21) were independent predictors of CIP. The nomogram demonstrated good discrimination (AUC 0.819, 95% CI 0.752–0.891) and calibration, with a bootstrap-corrected C-index of 0.751. Subgroup analyses showed consistent associations across immune checkpoint inhibitor types and treatment lines.

**Conclusion:**

The developed nomogram, incorporating key clinical and psychological predictors, offers a practical tool for individualized risk assessment of CIP in advanced NSCLC patients, potentially guiding early intervention and improving immunotherapy safety.

## 1 Introduction

Advanced -small cell lung cancer (NSCLC) remains a leading cause of cancer-related mortality worldwide despite significant advances in therapeutic modalities. In recent years, immune checkpoint inhibitors (ICIs) have revolutionized the treatment landscape of advanced NSCLC by enhancing antitumor immunity and offering improved survival outcomes. However, the clinical utility of ICIs is frequently complicated by immune-related adverse events (irAEs), among which checkpoint inhibitor-related pneumonitis (CIP) represents a particularly challenging and potentially life-threatening complication (1–3). The heterogeneity in clinical presentation and severity of IRP underscores the necessity for precise risk stratification and early identification of patients at heightened risk. The pathophysiology of IRP is not fully elucidated, but it is postulated to involve an aberrant immune response that disrupts pulmonary immune homeostasis, leading to inflammatory damage of lung parenchyma. Preclinical studies have suggested that dysregulation of T-cell mediated responses, enhanced cytokine release, and off-target effects on pulmonary tissue contribute to the development of CIP. Clinically, the diagnosis of CIP is predominantly based on radiographic imaging and exclusion of alternative etiologies; however, the non-specific radiological features and overlap with other pulmonary conditions often pose diagnostic challenges (4, 5).

Several clinical and biological factors have been implicated in increasing the susceptibility to CIP among NSCLC patients receiving ICIs. These include patient-specific variables such as age, smoking history, preexisting pulmonary conditions, and previous thoracic radiotherapy, as well as tumor-specific factors and immune profiles. Nonetheless, the predictive accuracy of these individual factors remains limited when used in isolation. Consequently, there is an emerging interest in developing comprehensive predictive models that integrate multiple risk factors to facilitate more accurate risk stratification. In this context, the development of a nomogram prediction model represents a promising approach for individualized risk assessment of CIP (6, 7). Nomograms have been widely adopted in oncology for their ability to incorporate heterogeneous prognostic variables and generate personalized risk estimates. By integrating clinical, radiological, and laboratory parameters, a nomogram could provide clinicians with a pragmatic tool to predict the likelihood of CIP, thereby enabling early intervention and optimization of management strategies. Furthermore, the use of such models may assist in balancing the therapeutic benefits of ICIs with the potential risks, ultimately contributing to improved patient outcomes (8–10).

The objective of this study is to identify the key risk factors associated with the development of CIP in patients with advanced NSCLC undergoing ICI therapy and to construct a validated nomogram prediction model. The findings from this investigation are expected to inform risk mitigation strategies, guide surveillance protocols, and ultimately contribute to the refinement of immunotherapeutic regimens in advanced NSCLC.

## 2 Materials and methods

### 2.1 Study design

This retrospective study identified consecutive patients with advanced NSCLC who received immune checkpoint inhibitor (ICI) therapy at our institution between January 2021 and December 2024. For prediction model development, a propensity score–matched analytic cohort was constructed. All patients who developed checkpoint CIP during follow-up were included as cases (*n* = 96). From the source population of patients without CIP (*n* = 856), a subset of non-CIP controls was selected using propensity score matching, yielding 191 matched controls. The study design, methodological framework, and analytical protocols were developed in accordance with the Strengthening the Reporting of Observational Studies in Epidemiology (STROBE) guidelines (11). Informed consent was obtained from all participants. The study was approved by the hospital’s ethics committee and conducted in accordance with relevant guidelines and the Declaration of Helsinki. All data were anonymized prior to analysis to ensure participant confidentiality.

### 2.2 Inclusion and exclusion criteria

The study enrolled patients based on the following inclusion criteria: (1) a histologically confirmed diagnosis of advanced NSCLC; (2) administration of immune checkpoint inhibitor therapy at our institution between January 2021 and December 2024; (3) age 18 years or older; (4) availability of comprehensive clinical records and adequate follow-up information; and (5) a minimum follow-up duration of 3 months to reliably assess the development of immune-related pneumonitis.

Patients were excluded if they met any of the following criteria: (1) a history of other active malignancies, except for those with a completely resected localized cancer with a disease-free interval of at least 5 years; (2) preexisting or concurrent autoimmune disorders that might confound the diagnosis of immune-related pneumonitis; (3) receipt of concurrent treatment modalities, such as chemotherapy or radiotherapy, that could interfere with the assessment of immune checkpoint inhibitor-induced adverse events.

### 2.3 Diagnostic criteria for CIP and exclusion of alternative etiologies

Checkpoint inhibitor–related pneumonitis was defined as a new inflammatory lung injury temporally associated with immune checkpoint inhibitor exposure. The diagnosis required new or worsening respiratory symptoms and/or newly identified radiographic abnormalities, together with new pulmonary infiltrates on chest computed tomography compatible with pneumonitis, including ground-glass opacities, consolidations, interstitial changes, or an organizing pneumonia–like pattern. Suspected events were initially evaluated by the treating oncology team, and cases with diagnostic uncertainty were jointly reviewed with respiratory medicine and radiology specialists. Clinical improvement following immunosuppressive therapy was regarded as supportive evidence but was not used as a mandatory diagnostic criterion.

Before classification as checkpoint inhibitor–related pneumonitis, a structured differential diagnostic process was applied to systematically exclude alternative etiologies. Infectious pneumonia was assessed based on clinical course, physical examination, laboratory testing (including complete blood count with differential and inflammatory biomarkers), and microbiological investigations when clinically indicated, such as respiratory specimen testing and blood cultures in febrile or severe cases. Radiation pneumonitis was evaluated in patients with a history of thoracic radiotherapy by correlating treatment timing with the spatial distribution of pulmonary abnormalities; infiltrates predominantly confined to irradiated fields and temporally consistent with radiation injury were not classified as CIP. Tumor progression or malignant pulmonary involvement was excluded through longitudinal imaging review and clinical correlation, including assessment for lymphangitic carcinomatosis or airway obstruction–related atelectasis. Cardiogenic causes of pulmonary infiltrates were explicitly considered during the adjudication process, particularly in patients presenting with dyspnea and diffuse ground-glass opacities. Cardiac etiologies, including heart failure and immune-related myocarditis, were evaluated based on cardiovascular history, physical examination findings, and ancillary cardiac investigations when clinically indicated, including electrocardiography, cardiac biomarkers, echocardiography, and natriuretic peptides (BNP/NT-proBNP). Imaging patterns and clinical features consistent with cardiogenic pulmonary edema, or symptom resolution following cardiac-directed therapy, were not classified as checkpoint inhibitor–related pneumonitis. Cases in which cardiac dysfunction or volume overload was considered the primary driver of symptoms and radiographic abnormalities were excluded from CIP classification, thereby minimizing diagnostic overlap and misclassification.

### 2.4 Data collection

Clinical data were retrospectively collected for both study groups. The variables included age, gender, disease duration (defined as the time interval from the initial pathological diagnosis of lung cancer to the initiation of immune checkpoint inhibitor therapy), pathological subtype, tumor diameter, history of thoracic radiotherapy, preexisting pulmonary diseases, smoking history, family history of pneumonia, body mass index (kg/m^2^), anxiety status, psychological resilience, and quality of life. Anxiety severity was evaluated using the Hamilton Anxiety Rating Scale (HAMA), which comprises 14 items scored on a five-point Likert scale ranging from 0 (“none”) to 4 (“extremely severe”), with higher scores indicating more pronounced anxiety. The HAMA has demonstrated satisfactory reliability, with a Cronbach’s alpha coefficient of 0.816. Psychological resilience was assessed using the abbreviated 10-item Connor-Davidson Resilience Scale (CD-RISC 10). Each item is scored from 0 to 4, yielding a total score range of 0–40, where higher scores reflect greater resilience. This instrument has shown good reliability, with a Cronbach’s alpha coefficient of 0.791. Quality of life was measured using the QLQ-C30, a validated instrument that evaluates five dimensions: cognitive, physical, social, and emotional functioning, as well as global health status. The QLQ-C30 generates a composite score ranging from 0 to 100, with higher scores indicating better quality of life.

### 2.5 Follow-up procedures

Patients were monitored through outpatient visits, telephone calls, and WeChat communications. The follow-up period extended until 31 March 2025. The primary observation endpoint was the occurrence of CIP in patients with advanced non-small cell lung cancer, and the time of CIP onset was systematically recorded. The diagnosis of CIP was established based on a combination of clinical, radiological, and laboratory assessments. Specifically, CIP was considered when patients presented with new-onset respiratory symptoms, such as cough, dyspnea, and fever, along with radiological findings on chest computed tomography (CT) indicative of inflammatory changes, including ground-glass opacities and consolidations, that were not attributable to alternative causes. Differential diagnosis required the exclusion of infectious etiologies, disease progression, and other non-immunotherapy related pulmonary conditions. In addition, a favorable response to immunosuppressive treatment was regarded as supportive evidence for CIP. These diagnostic criteria were formulated in accordance with established clinical guidelines and consensus recommendations to ensure a standardized and objective assessment of CIP (12).

### 2.6 Statistical analysis

Statistical analyses were conducted using SPSS version 26.0 and R version 4.3.3. Continuous variables were summarized as mean ± standard deviation, and differences between groups were evaluated using the independent-samples *t*-test. Categorical data were expressed as counts (percentages), and group comparisons were performed utilizing the chi-square test, continuity-corrected chi-square test, or Fisher’s exact test, as appropriate. Variables demonstrating statistical significance in the univariate analysis were subsequently incorporated into a multivariate logistic regression model to identify independent risk factors. The rms package in R was employed to construct a nomogram for predicting the risk of CIP. The discriminative performance of the model was assessed by generating receiver operating characteristic (ROC) curves. The optimal cut-off value for the prediction model was determined by maximizing the Youden index, thereby providing estimates of the model’s sensitivity and specificity. Model calibration was evaluated using the Hosmer–Lemeshow goodness-of-fit test. Internal validation was performed through bootstrap resampling (*n* = 1,000), which yielded a corrected C-index and allowed the generation of a calibration curve to illustrate the nomogram’s predictive accuracy. Additionally, decision curve analysis (DCA) was implemented to assess the clinical utility of the predictive model. To explore potential clinical heterogeneity, prespecified subgroup analyses were performed according to immune checkpoint inhibitor type (PD-1 vs. PD-L1 inhibitors), treatment line (first-line vs. later-line therapy), and major subtypes of underlying lung disease. Given the limited sample size within individual strata, these analyses were considered exploratory. Interaction terms between subgroup variables and the main predictors in the final multivariable model were introduced into logistic regression models to assess effect modification. A non-significant interaction *P*-value (>0.05) was interpreted as no evidence of heterogeneity across subgroups. A *P*-value of less than 0.05 was considered indicative of statistical significance.

## 3 Results

### 3.1 Baseline clinical characteristics and their associations with the development of CIP in patients with advanced NSCLC

Within the propensity score–matched analytic cohort (*n* = 287), 96 patients were classified as having developed CIP and 191 were matched non-CIP controls. The CIP group exhibited a higher mean age (66.100 ± 6.400 years) compared to the non-CIP group (59.900 ± 5.200 years; *P* < 0.001) and demonstrated a longer disease duration (6.300 ± 1.500 versus 5.500 ± 1.200 years; *P* < 0.001). Additionally, patients with CIP presented with significantly elevated HAMA scores (31.400 ± 6.500 points versus 27.300 ± 5.100 points; *P* < 0.001), indicative of higher anxiety levels. Baseline spirometric parameters, including forced expiratory volume in 1 s (FEV1) and forced vital capacity (FVC), were comparable between patients with and without CIP. Although no significant differences were noted in BMI, tumor diameter, CD-RISC-10, or QLQ-C30 scores, categorical analyses revealed a markedly higher prevalence of smoking history in the CIP group (68.800% versus 28.800%; *P* < 0.001). Moreover, prior thoracic radiotherapy and underlying lung disease were significantly more common in patients with CIP (64.600% versus 28.300% and 65.600% versus 38.200%, respectively; both *P* < 0.001). A family history of pneumonia was also more frequently reported among CIP patients (56.300% versus 35.600%; *P* = 0.013) (Table 1).

**TABLE 1:** Baseline clinical characteristics of patients with advanced non-small cell lung cancer (NSCLC) stratified by checkpoint inhibitor-related pneumonitis (CIP) status.

Variable	Pneumonitis group (*n* = 96)	Non-pneumonitis group (*n* = 191)	Statistic	*P*-value
Age (years)	66.1 ± 6.4	59.9 ± 5.2	*t* = 6.42	<0.001
BMI (kg/m^2^)	23.3 ± 2.4	23.5 ± 2.3	*t* = 0.80	0.425
Disease duration (years)	6.3 ± 1.5	5.5 ± 1.2	*t* = 4.00	<0.001
Tumor diameter (cm)	5.3 ± 1.1	5.0 ± 1.1	*t* = 1.45	0.151
HAMA score (points)	31.4 ± 6.5	27.3 ± 5.1	*t* = 4.21	<0.001
CD-RISC-10 score (points)	26.8 ± 4.7	26.0 ± 4.9	*t* = 0.95	0.342
QLQ-C30 score (points)	76.9 ± 14.4	79.2 ± 12.5	*t* = 1.05	0.297
Sex, *n* (%)			χ^2^ = 0.071	0.789
- Male	68 (70.8%)	133 (69.6%)		
- Female	28 (29.2%)	58 (30.4%)		
FEV1 (L)	1.89 ± 0.76	2.02 ± 0.31	*t* = 1.61	0.110
FVC (L)	2.88 ± 0.65	3.01 ± 0.72	*t* = 1.54	0.125
Smoking history, *n* (%)			χ^2^ = 22.94	< 0.001
- Yes	66 (68.8%)	55 (28.8%)		
- No	30 (31.2%)	136 (71.2%)		
Pathological type, *n* (%)			χ^2^ = 3.08	0.223
- Adenocarcinoma	27 (28.1%)	71 (37.2%)		
- Squamous carcinoma	38 (39.6%)	52 (27.2%)		
- Large cell carcinoma	30 (31.3%)	67 (35.1%)		
History of thoracic radiotherapy, *n* (%)			χ^2^ = 21.01	<0.001
- Yes	62 (64.6%)	54 (28.3%)		
- No	34 (35.4%)	137 (71.7%)		
History of underlying lung disease, *n* (%)			χ^2^ = 11.55	<0.001
- Yes	63 (65.6%)	73 (38.2%)		
- No	33 (34.4%)	118 (61.8%)		
Family history of pneumonia, *n* (%)			χ^2^ = 6.23	0.013
- Yes	54 (56.3%)	68 (35.6%)		
- No	42 (43.7%)	123 (64.4%)		

BMI, body mass index; HAMA, Hamilton Anxiety Rating Scale; CD-RISC-10, 10-item Connor-Davidson Resilience Scale; QLQ-C30, Quality of Life Questionnaire Core 30.

### 3.2 Multivariate analysis of independent predictors for CIP in advanced NSCLC

Multivariate logistic regression analysis was conducted to determine independent predictors for the development of CIP in advanced NSCLC patients. Disease duration was significantly associated with CIP, with a regression coefficient of 0.508 (SE = 0.170), corresponding to an OR of 1.660 (95% CI: 1.195–2.310; *P* = 0.003). Smoking history also emerged as an independent predictor (regression coefficient: 1.210; SE = 0.468; OR: 3.320; 95% CI: 1.340–8.260; *P* = 0.017). Furthermore, a prior history of chest radiotherapy was significantly correlated with CIP (regression coefficient: 1.005; SE = 0.470; OR: 2.750; 95% CI: 1.090–6.920; *P* = 0.032). Elevated HAMA scores were significantly linked to CIP risk (regression coefficient: 0.116; SE = 0.038; OR: 1.124; 95% CI: 1.043–1.209; *P* = 0.002). In contrast, age, underlying lung disease history, and family history of pneumonia did not show statistically significant associations with CIP (*P* > 0.05) (Table 2).

**TABLE 2:** Multivariate logistic regression analysis of independent predictors for CIP in patients with advanced NSCLC.

Independent variable	Regression coefficient	Standard error	Wald X^2^ value	OR value	95% CI	*P*-value
Age	0.017	0.033	0.265	1.018	[0.956, 1.084	0.617
Underlying lung disease history	0.293	0.445	0.433	1.338	[0.560, 3.210]	0.516
Family history of pneumonia	0.124	0.475	0.068	1.132	[0.447, 2.870]	0.795
Disease duration	0.508	0.17	8.93	1.66	[1.195, 2.310]	0.003
Smoking history	1.21	0.468	6.685	3.32	[1.340, 8.260]	0.017
Chest radiotherapy History	1.005	0.47	4.577	2.75	[1.090, 6.920]	0.032
HAMA score	0.116	0.038	9.321	1.124	[1.043, 1.209]	0.002

HAMA, Hamilton Anxiety Rating Scale; CI, confidence interval; OR, odds ratio; CIP, checkpoint inhibitor-related pneumonitis; NSCLC, non-small cell lung cancer.

### 3.3 Establishment and validation of the CIP nomogram predictive model

**FIGURE 1:**
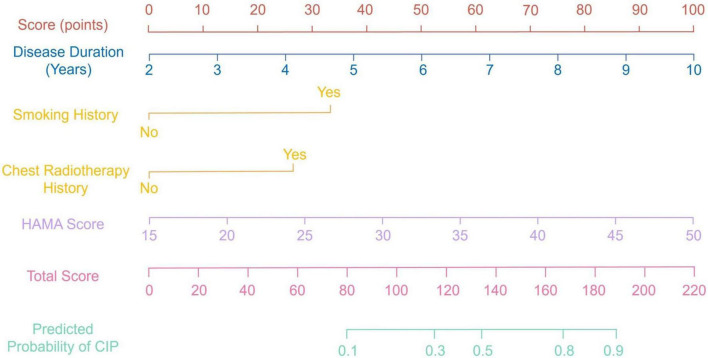
Nomogram for predicting the risk of checkpoint inhibitor-related pneumonitis in advanced non-small cell lung cancer patients.

**FIGURE 2:**
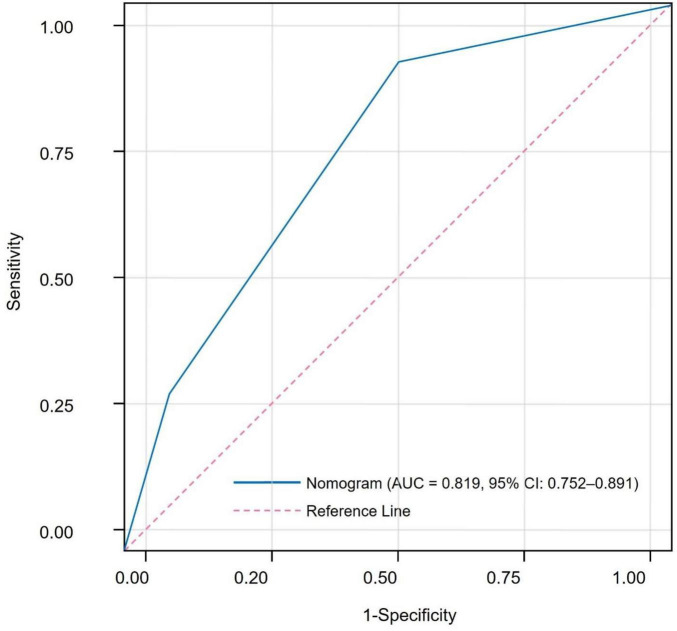
Receiver operating characteristic (ROC) curve demonstrating the discriminative ability of the nomogram for predicting checkpoint inhibitor-related pneumonitis.

**FIGURE 3:**
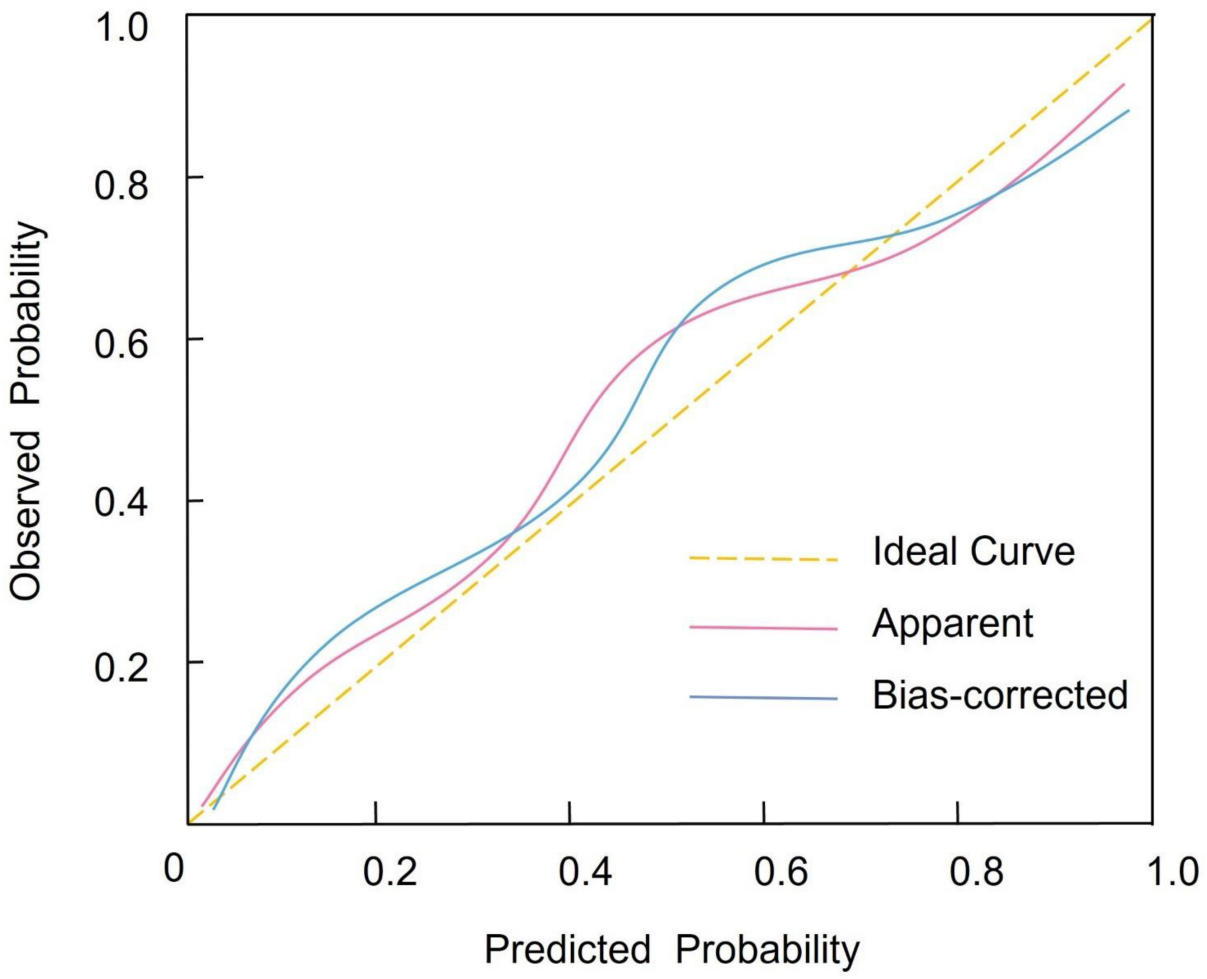
Calibration curve depicting the agreement between predicted probabilities and observed outcomes of checkpoint inhibitor-related pneumonitis as estimated by the nomogram.

**FIGURE 4:**
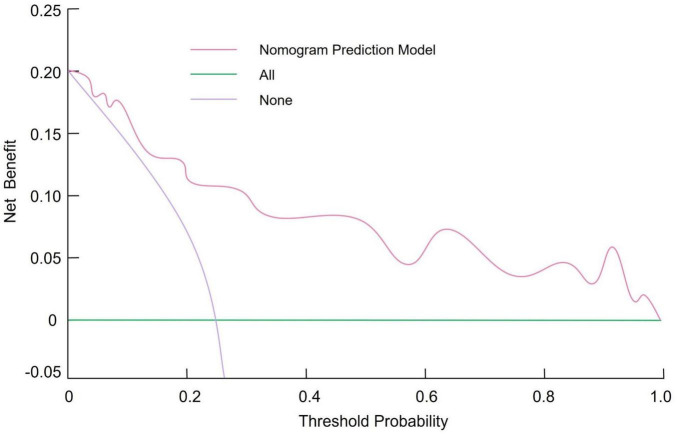
Decision curve analysis (DCA) illustrating the net clinical benefit of the nomogram for predicting checkpoint inhibitor-related pneumonitis.

Based on multivariate logistic regression analysis, four independent predictors were selected to construct a nomogram for predicting the risk of CIP in advanced NSCLC patients. In this model, each predictor is represented by a point scale along its respective axis. The individual points corresponding to each variable are summed to yield a total score, which is then mapped to a probability scale indicating the likelihood of CIP occurrence. Higher total scores are associated with an increased risk of developing CIP (Figure 1). The discriminative performance of the nomogram was assessed by calculating the AUC, which was found to be 0.819 (95% CI: 0.752–0.891). At the optimal cutoff determined by the maximum Youden index, the sensitivity and specificity were 75.92% and 83.65%, respectively (Figure 2). Internal validation using bootstrap resampling (n = 1,000) yielded a corrected C-index of 0.751 (95% CI: 0.705–0.812). The Hosmer–Lemeshow goodness-of-fit test further confirmed the model’s calibration (χ^2^ = 2.167, *P* = 0.863), with the calibration curve demonstrating a close agreement between predicted and observed probabilities (Figure 3). Additionally, DCA revealed that the net benefit of the nomogram substantially exceeded that of the extreme strategies, underscoring its clinical utility in guiding CIP risk management (Figure 4).

### 3.4 Subgroup analyses

Exploratory subgroup analyses were performed to evaluate the consistency of the associations between the identified risk factors and the development of checkpoint inhibitor-related pneumonitis across clinically relevant strata. The direction and magnitude of associations for disease duration, smoking history, prior chest radiotherapy, and HAMA score were generally consistent across subgroups defined by immune checkpoint inhibitor type (PD-1 vs. PD-L1 inhibitors), treatment line (first-line vs. later-line therapy), and major categories of underlying lung disease. No statistically significant interaction effects were observed (all interaction *P*-values > 0.05), suggesting no strong evidence of effect modification. Owing to the exploratory nature of these analyses and the limited number of patients within certain subgroups, the results should be interpreted with caution. Detailed subgroup distributions and interaction testing results are provided in Supplementary Table 1.

### 3.5 *Post hoc* power analysis

A *post hoc* power analysis was performed to evaluate the adequacy of the sample size for detecting the associations identified in the multivariable logistic regression model. The analysis was based on the four independent predictors retained in the final model, disease duration, smoking history, prior chest radiotherapy, and HAMA score. Assuming equal contribution of each predictor to the overall model, the weighted overall *post hoc* statistical power was estimated to be 84.03%, exceeding the commonly accepted threshold of 80%. These findings suggest that the study was sufficiently powered to detect the observed independent associations with the risk of checkpoint inhibitor–related pneumonitis.

## 4 Discussion

In this study, we investigated the risk factors for CIP in patients with advanced NSCLC undergoing immunotherapy and developed a nomogram to predict its occurrence. Our analysis of 287 patients revealed significant differences in baseline clinical characteristics between those who developed CIP and those who did not. The CIP group was characterized by higher mean age, prolonged disease duration, and elevated anxiety levels. In addition, a significantly higher prevalence of smoking history, prior thoracic radiotherapy, underlying lung disease, and family history of pneumonia was observed in the CIP group. These findings suggest that both intrinsic patient factors and treatment-related variables contribute to the development of CIP. Multivariate logistic regression identified four independent predictors for CIP: disease duration, smoking history, prior chest radiotherapy, and HAMA score. Specifically, prolonged disease duration was associated with a 66% increased risk of CIP, while smoking history and prior chest radiotherapy conferred a 3-fold and 2.75-fold increase in risk, respectively. The association between elevated HAMA scores and CIP risk suggests a potential role for psychological factors in modulating immune responses during therapy. Based on these predictors, we constructed a nomogram that demonstrated robust discrimination (AUC = 0.819) and good calibration (Hosmer–Lemeshow *P* = 0.863, corrected C-index = 0.751). Decision curve analysis further supported the clinical utility of the nomogram by indicating a net benefit over alternative strategies.

The results of our study are consistent with and extend previous findings in the literature on immunotherapy-associated pulmonary toxicity in NSCLC. Prior studies have documented that advanced age and prolonged disease duration are risk factors for CIP, likely reflecting cumulative treatment exposure and the reduced pulmonary reserve seen in older patients. In our cohort, while age showed significant differences in univariate analysis, it did not emerge as an independent predictor after multivariate adjustment. This suggests that the effect of age may be mediated by other comorbidities or treatment exposures, such as smoking and prior chest radiotherapy. Smoking history was a strong independent predictor in our analysis (13, 14). This aligns with previous research indicating that smoking-induced lung injury predisposes patients to inflammatory complications during immunotherapy. Likewise, the association between a history of chest radiotherapy and CIP corroborates earlier findings that prior lung irradiation can compromise lung integrity, thereby increasing susceptibility to immune-mediated damage (15, 16). The novel finding in our study is the independent association of elevated HAMA scores with CIP risk. Although limited data exist regarding the impact of psychological stress on immune-mediated adverse events, our findings support the hypothesis that heightened anxiety may contribute to a dysregulated immune response. This observation underscores the need to consider psychosocial factors when assessing patient risk profiles for CIP (17–19).

Overall, our findings emphasize the multifactorial etiology of CIP and highlight the interplay between clinical, treatment-related, and psychosocial factors. Compared to prior studies, our work uniquely integrates an anxiety measure into the predictive model, potentially offering a more comprehensive risk assessment framework for clinicians. The development of a nomogram incorporating these variables provides a practical tool that enhances individualized risk stratification and may facilitate timely intervention to mitigate the severity of CIP. Such integrated risk models are increasingly recognized as essential in tailoring immunotherapeutic strategies, and our study contributes to this evolving paradigm by validating the prognostic significance of multiple risk factors in a real-world NSCLC population.

The identification of independent predictors for CIP has significant clinical implications. Clinicians can utilize the nomogram to stratify advanced NSCLC patients based on their individual risk of developing CIP, facilitating personalized monitoring and management. Patients identified as high-risk, those with longer disease duration, positive smoking history, prior chest radiotherapy, and elevated anxiety levels, should be closely monitored during immunotherapy. Early detection and intervention in these patients may prevent progression to severe pneumonitis and improve overall treatment outcomes. Moreover, these findings highlight the importance of addressing modifiable risk factors, such as smoking cessation and anxiety management, as part of comprehensive cancer care (20–22).

A major strength of this study is the comprehensive evaluation of both clinical and psychosocial factors in a well-defined cohort of advanced NSCLC patients. The integration of traditional risk factors (such as smoking history and prior chest radiotherapy) with a psychosocial measure (HAMA score) represents a novel approach in predicting CIP risk. The development of a nomogram based on multivariate logistic regression further enhances the clinical utility of our findings by providing an individualized risk assessment tool. Additionally, internal validation using bootstrap resampling and the use of robust statistical methods, including ROC analysis, calibration curves, and decision curve analysis, support the reliability and potential clinical applicability of the model. Several limitations of this study should be acknowledged. First, the retrospective single-center design may introduce selection bias and limit generalizability. Although internal bootstrap validation demonstrated acceptable discrimination and calibration, the absence of external validation restricts the transportability of the nomogram across different clinical settings. Therefore, the model should be regarded as an exploratory and hypothesis-generating risk stratification tool rather than a universally applicable prediction model, and external validation in independent multicenter cohorts is warranted. Second, treatment-related heterogeneity may influence the risk of checkpoint inhibitor-related pneumonitis; however, further stratification by specific immune checkpoint inhibitor agents, combination regimens, or Cytotoxic T-Lymphocyte–Associated Protein 4 (CTLA-4) based therapies was limited by small subgroup sample sizes and could have compromised model stability. Larger multicenter studies are needed to enable treatment-specific risk modeling. Third, although the Hamilton Anxiety Rating Scale emerged as an independent predictor, it should not be interpreted as a causal biological driver of immune-mediated lung injury. The HAMA score was incorporated as a baseline risk-associated variable for prediction, and residual confounding inherent to the retrospective design cannot be fully excluded. Moreover, its routine use in oncological practice is not universal and requires further validation. Finally, detailed data on concurrent medications and genetic or molecular biomarkers were not consistently available in this real-world cohort and could not be incorporated into the model. Future prospective studies with standardized data collection, including medication exposure and biomarker profiling, are needed to further enhance predictive accuracy and biological interpretability.

## 5 Conclusion

This study identified prolonged disease duration, smoking history, prior chest radiotherapy, and elevated HAMA scores as independent predictors for CIP in advanced NSCLC patients. A nomogram based on these factors demonstrated robust predictive accuracy and calibration. These findings offer a practical tool for individualized risk assessment and early intervention to optimize immunotherapy safety.

## Data Availability

The raw data supporting the conclusions of this article will be made available by the authors, without undue reservation.

## References

[1] Kim S Lim J. Immune checkpoint inhibitor-related interstitial lung disease in patients with advanced non-small cell lung cancer: systematic review of characteristics, incidence, risk factors, and management. *J Thorac Dis.* (2022) 14:1684–95. 10.21037/jtd-22-93 35693611 PMC9186237

[2] Yamagata A Yokoyama T Fukuda Y Ishida T. Impact of interstitial lung disease associated with immune checkpoint inhibitors on prognosis in patients with non-small-cell lung cancer. *Cancer Chemother Pharmacol.* (2021) 87:251–8. 10.1007/s00280-020-04205-x 33394102

[3] Hao Y Zhang X Yu L. Immune checkpoint inhibitor-related pneumonitis in non-small cell lung cancer: a review. *Front Oncol.* (2022) 12:911906. 10.3389/fonc.2022.911906 36052257 PMC9424849

[4] Atchley W Alvarez C Saxena-Beem S Schwartz T Ishizawar R Patel K et al. Immune checkpoint inhibitor-related pneumonitis in lung cancer: real-world incidence, risk factors, and management practices across six health care centers in North Carolina. *Chest.* (2021) 160:731–42. 10.1016/j.chest.2021.02.032 33621599 PMC8411447

[5] Sternschein R Moll M Ng J D’Ambrosio C. Immune checkpoint inhibitor-related pneumonitis. Incidence, risk factors, and clinical and radiographic features. *Am J Respir Crit Care Med.* (2018) 198:951–3. 10.1164/rccm.201803-0525RR 30095979 PMC6835075

[6] Liu X Hao N Yang S Li J Wang L. Predictive factors and prognosis of immune checkpoint inhibitor-related pneumonitis in non-small cell lung cancer patients. *Front Oncol.* (2023) 13:1145143. 10.3389/fonc.2023.1145143 37182127 PMC10169751

[7] Huang A Xu Y Zang X Wu C Gao J Sun X et al. Radiographic features and prognosis of early- and late-onset non-small cell lung cancer immune checkpoint inhibitor-related pneumonitis. *BMC Cancer.* (2021) 21:634. 10.1186/s12885-021-08353-y 34051746 PMC8164260

[8] Zhang Y Zhang L Cao S Wang Y Ling X Zhou Y et al. A nomogram model for predicting the risk of checkpoint inhibitor-related pneumonitis for patients with advanced non-small-cell lung cancer. *Cancer Med.* (2023) 12:15998–6010. 10.1002/cam4.6244 37409360 PMC10469710

[9] Kikuchi R Watanabe Y Okuma T Nakamura H Abe S. Outcome of immune checkpoint inhibitor treatment in non-small cell lung cancer patients with interstitial lung abnormalities: clinical utility of subcategorizing interstitial lung abnormalities. *Cancer Immunol Immunother.* (2024) 73:211. 10.1007/s00262-024-03792-5 39235641 PMC11377385

[10] Sun N Li R Deng H Li Q Deng J Zhu Y et al. The prognostic impact of severe grade immune checkpoint inhibitor related pneumonitis in non-small cell lung cancer patients. *Front Oncol.* (2024) 14:1372532. 10.3389/fonc.2024.1372532 38983925 PMC11231069

[11] von Elm E Altman D Egger M Pocock S Gøtzsche P Vandenbroucke J. The Strengthening the reporting of observational studies in epidemiology (STROBE) statement: guidelines for reporting observational studies. *J Clin Epidemiol.* (2008) 61:344–9. 10.1016/j.jclinepi.2007.11.008 18313558

[12] Sun X Song Z Jiang H Ma Y Chen M. Image classification of immune checkpoint inhibitor-related pneumonia in lung cancer patients. *Clin Imaging.* (2022) 86:31–7. 10.1016/j.clinimag.2022.03.012 35325631

[13] Mao Z Pang G Huang X Chen X Wu J Xu X et al. Risk factors of immune checkpoint inhibitor-related pneumonitis after neoadjuvant immunochemotherapy for resectable NSCLC. *BMC Pulm Med.* (2024) 24:253. 10.1186/s12890-024-03041-6 38783253 PMC11112843

[14] Moda M Saito H Kato T Usui R Kondo T Nakahara Y et al. Tumor invasion in the central airway is a risk factor for early-onset checkpoint inhibitor pneumonitis in patients with non-small cell lung cancer. *Thorac Cancer.* (2020) 11:3576–84. 10.1111/1759-7714.13703 33078531 PMC7705619

[15] Cadranel J Canellas A Matton L Darrason M Parrot A Naccache J et al. Pulmonary complications of immune checkpoint inhibitors in patients with nonsmall cell lung cancer. *Eur Respir Rev.* (2019) 28:190058. 10.1183/16000617.0058-2019 31597674 PMC9488121

[16] Li Y Jiang Y Pan L Yao J Liang S Du Y et al. First-line chemoimmunotherapy for patients with small-cell lung cancer and interstitial lung abnormality: CIP risk and prognostic analysis. *Thorac Cancer.* (2024) 15:2437–48. 10.1111/1759-7714.15471 39435523 PMC11609049

[17] Yang J Lyu M Feng X Liu F Zeng R Sun X et al. The predict factors and clinical prognosis value of immune-related pneumonia of receiving PD-1 inhibitor in advanced non-small cell lung cancer: a retrospective study. *Int Immunopharmacol.* (2024) 142:113140. 10.1016/j.intimp.2024.113140 39312858

[18] Zhai X Zhang J Tian Y Li J Jing W Guo H et al. The mechanism and risk factors for immune checkpoint inhibitor pneumonitis in non-small cell lung cancer patients. *Cancer Biol Med.* (2020) 17:599–611. 10.20892/j.issn.2095-3941.2020.0102 32944393 PMC7476083

[19] Chu X Zhao J Zhou J Zhou F Jiang T Jiang S et al. Association of baseline peripheral-blood eosinophil count with immune checkpoint inhibitor-related pneumonitis and clinical outcomes in patients with non-small cell lung cancer receiving immune checkpoint inhibitors. *Lung Cancer.* (2020) 150:76–82. 10.1016/j.lungcan.2020.08.015 33080551

[20] Horiuchi K Ikemura S Sato T Shimozaki K Okamori S Yamada Y et al. Pre-existing interstitial lung abnormalities and immune checkpoint inhibitor-related pneumonitis in solid tumors: a retrospective analysis. *Oncologist.* (2024) 29:e108–17. 10.1093/oncolo/oyad187 37590388 PMC10769794

[21] Cui L Cheng K Cui M Li X. Characteristics and risk factors of immune checkpoint inhibitor-related pneumonitis in non-small cell lung cancer: a retrospective study. *Oncology.* (2025) 108:699–708. 10.1159/000543556 39929164

[22] Cho J Kim J Lee J Kim Y Kim S Lee Y et al. Characteristics, incidence, and risk factors of immune checkpoint inhibitor-related pneumonitis in patients with non-small cell lung cancer. *Lung Cancer.* (2018) 125:150–6. 10.1016/j.lungcan.2018.09.015 30429014

