# Supplementary material for: Risk factors and nomogram prediction model for checkpoint inhibitor-related pneumonitis in patients with advanced non-small cell lung cancer
Source: Front Med (Lausanne). 2026 Mar 25;13:1742594. doi: 10.3389/fmed.2026.1742594 (PMC13057278; doi:10.3389/fmed.2026.1742594)
Supplement: Supplementary file 1 [file Table_1.docx]

**Supplementary Table S1.** Subgroup analyses of risk factors for Checkpoint Inhibitor-related Pneumonitis

| Subgroup variable | n (CIP / non-CIP) | Direction of association | Interaction P |
| --- | --- | --- | --- |
| Immune checkpoint inhibitor type |  |  |  |
| PD-1 inhibitors | 68 / 142 | Consistent | 0.48 |
| PD-L1 inhibitors | 28 / 49 | Consistent | 0.61 |
| Treatment line |  |  |  |
| First-line ICI therapy | 52 / 108 | Consistent | 0.39 |
| Later-line ICI therapy | 44 / 83 | Consistent | 0.57 |
| Underlying lung disease subtype |  |  |  |
| COPD | 34 / 51 | Consistent | 0.44 |
| Interstitial lung disease (ILD) | 19 / 27 | Consistent | 0.52 |
| Other chronic lung diseases | 10 / 35 | Consistent | 0.63 |

“Consistent” indicates that the direction of association for the main predictors (disease duration, smoking history, prior chest radiotherapy, and HAMA score) was concordant with the overall model estimates. Interaction P values were derived from logistic regression models including interaction terms between subgroup variables and the main predictors.

Abbreviations: CIP, checkpoint inhibitor–related pneumonitis; ICI, immune checkpoint inhibitor; PD-1, programmed cell death protein 1; PD-L1, programmed death-ligand 1; COPD, chronic obstructive pulmonary disease; ILD, interstitial lung disease; HAMA, Hamilton Anxiety Rating Scale.
